# Quantitative multiparametric MRI predicts response to neoadjuvant therapy in the community setting

**DOI:** 10.1186/s13058-021-01489-6

**Published:** 2021-11-27

**Authors:** John Virostko, Anna G. Sorace, Kalina P. Slavkova, Anum S. Kazerouni, Angela M. Jarrett, Julie C. DiCarlo, Stefanie Woodard, Sarah Avery, Boone Goodgame, Debra Patt, Thomas E. Yankeelov

**Affiliations:** 1 Department of Diagnostic Medicine, University of Texas at Austin, Austin, TX 78712 USA; 2 Livestrong Cancer Institutes, University of Texas at Austin, Austin, TX USA; 3 Department of Oncology, University of Texas at Austin, Austin, TX USA; 4 Oden Institute for Computational Engineering and Sciences, University of Texas at Austin, Austin, TX USA; 5 Department of Radiology, University of Alabama at Birmingham, Birmingham, AL USA; 6 Department of Biomedical Engineering, University of Alabama at Birmingham, Birmingham, AL USA; 7 O’Neal Comprehensive Cancer Center, University of Alabama at Birmingham, Birmingham, AL USA; 8 Department of Physics, University of Texas at Austin, Austin, TX USA; 9 Department of Radiology, University of Washington, Seattle, WA USA; 10 Austin Radiological Association, Austin, TX USA; 11 Dell Seton Medical Center at the University of Texas, Austin, USA; 12 Texas Oncology, Austin, TX USA; 13 Department of Biomedical Engineering, University of Texas at Austin, Austin, TX USA; 14 Department of Imaging Physics, MD Anderson Cancer Center, Houston, TX USA

**Keywords:** NAT, Diffusion, Dynamic contrast enhanced, DW-MRI, DCE-MRI

## Abstract

**Background:**

The purpose of this study was to determine whether advanced quantitative magnetic resonance imaging (MRI) can be deployed outside of large, research-oriented academic hospitals and into community care settings to predict eventual pathological complete response (pCR) to neoadjuvant therapy (NAT) in patients with locally advanced breast cancer.

**Methods:**

Patients with stage II/III breast cancer (*N* = 28) were enrolled in a multicenter study performed in community radiology settings. Dynamic contrast-enhanced (DCE) and diffusion-weighted (DW)-MRI data were acquired at four time points during the course of NAT. Estimates of the vascular perfusion and permeability, as assessed by the volume transfer rate (*K*^trans^) using the Patlak model, were generated from the DCE-MRI data while estimates of cell density, as assessed by the apparent diffusion coefficient (ADC), were calculated from DW-MRI data. Tumor volume was calculated using semi-automatic segmentation and combined with *K*^trans^ and ADC to yield bulk tumor blood flow and cellularity, respectively. The percent change in quantitative parameters at each MRI scan was calculated and compared to pathological response at the time of surgery. The predictive accuracy of each MRI parameter at different time points was quantified using receiver operating characteristic curves.

**Results:**

Tumor size and quantitative MRI parameters were similar at baseline between groups that achieved pCR (*n* = 8) and those that did not (*n* = 20). Patients achieving a pCR had a larger decline in volume and cellularity than those who did not achieve pCR after one cycle of NAT (*p* < 0.05). At the third and fourth MRI, changes in tumor volume, *K*^trans^, ADC, cellularity, and bulk tumor flow from baseline (pre-treatment) were all significantly greater (*p* < 0.05) in the cohort who achieved pCR compared to those patients with non-pCR.

**Conclusions:**

Quantitative analysis of DCE-MRI and DW-MRI can be implemented in the community care setting to accurately predict the response of breast cancer to NAT. Dissemination of quantitative MRI into the community setting allows for the incorporation of these parameters into the standard of care and increases the number of clinical community sites able to participate in novel drug trials that require quantitative MRI.

**Supplementary Information:**

The online version contains supplementary material available at 10.1186/s13058-021-01489-6.

## Introduction

Quantitative imaging allows for the characterization of biological phenomena from radiological data and has developed to the point where it is regularly incorporated in oncology clinical trials in the academic setting [1, 2]; however, it has yet to be expanded into the traditional radiology setting. With 85% of oncology patients receiving care and imaging from local or regional clinics [3], there is a need to move these advancements from academic institutes into community care facilities. Quantitative MRI has shown particular promise in predicting the response of breast tumors to neoadjuvant therapy (NAT) [4–6]. NAT, administration of therapy prior to definitive surgical resection of disease, is the standard-of-care approach for patients with locally advanced breast cancer (i.e., stage II–III). The main objectives of NAT are to improve overall patient survival by: (1) reducing the primary tumor burden for surgical resection, (2) treating clinically occult micrometastases, and (3) evaluating the impact of systemic therapies on breast cancer biology to improve selection of downstream therapeutic regimens [7–13]. Patients who achieve a pathological complete response (pCR, i.e., absence of viable tumor cells in the primary or local lymph nodes) following NAT have shown improved outcomes with increased survival rates [7, 8]. Conversely, patients with residual disease (i.e., non-pCR) at the conclusion of NAT have an increased risk of early recurrence and poorer prognoses [14–18]. Thus, accurate and early assessment of response to NAT would provide the opportunity to replace an ineffective treatment with an alternative regimen, potentially improving outcomes while simultaneously avoiding or decreasing side effects of ineffective therapies. As quantitative MRI has been shown to predict the response of breast tumors early in the course of NAT in academic settings [19, 20], a natural progression toward implementation into standard of care is to integrate such techniques into both multi-site and community-based settings. As the majority of cancer patients receive their care in the community setting [21], this will allow for improved care for a dramatically large percentage of breast cancer patients.

Evaluating early changes in cellularity and vascularity through quantitative MRI provides the opportunity to obtain serial three-dimensional biological characterization of the tumor at baseline and in response to systemic treatments. Diffusion-weighted (DW)-MRI and dynamic contrast-enhanced (DCE)-MRI have the potential to characterize spatial and temporal alterations in breast cancer cells and the tumor microenvironment prior to downstream effects of changes in tumor size. Tracking alterations in the apparent diffusion coefficient (ADC), extracted from DW-MRI, has been used to predict the response of breast tumors to therapy [22–24]. The ADC correlates with cellularity [25–27] and may be a better predictor of eventual response than measurements of tumor size [28]. DCE-MRI pharmacokinetic parameters present quantitative information relating to the changes in vascular delivery and extracellular space and have been demonstrated to be predictive of pCR in breast tumors undergoing NAT [19, 23, 29, 30]. DCE-MRI parameters such as *K*^trans^ (the volume transfer rate, related to vascular permeability and perfusion) can provide information on tumor vascularity. These emerging metrics have also shown clinical promise at informing advanced mathematical models that further describe the underlying cellular and biological features of the tumor during therapy [31, 32]. While biopsy samples are an integral component of breast cancer diagnosis and treatment guidance, imaging provides a powerful and complementary, noninvasive tool to probe the entire tumor microenvironment. Advanced measurements quantifying underlying tumor biology represent a comprehensive and personalized approach to monitor and predict response in cancer care.

To maximize the impact of quantitative imaging of locally advanced breast cancer during NAT, quantitative imaging must be implemented and validated in the community setting. Further, these methods must be robust enough to be utilized in many different breast cancer subtypes and across various sequencing, timing, and types of systemic therapy. We have previously shown that quantitative MRI metrics (ADC from DW-MRI and *T*_1_ mapping required for DCE-MRI) can be extracted from imaging protocols deployed in the community setting on phantoms and normal subjects with high repeatability and reproducibility [33]. The present study seeks to employ these techniques in the community setting to prospectively predict the response of locally advanced breast cancer and disseminate quantitative MRI beyond academia and toward routine application in the standard-of-care setting.

## Methods

### Study population

Women (*N* = 28) who were previously diagnosed with locally advanced breast cancer (stages II–III) and prescribed NAT were enrolled into this prospective clinical imaging study. The median age of patients was 44.5 years old (range of 25–74 years). See Table 1 for a summary of patient demographics and treatment regimens. Treatment was selected by the patient’s oncologist prior to study enrollment. No hormonal therapy was performed in this population.

**Table 1:** Clinical features of the study population (pathological complete response, pCR)

Patient #	Age [years]	ER/PR/HER2	Therapeutic regimen	Pathological response
1	54	+/−/−	doxorubicin/cyclophosphamide → paclitaxel	Non-pCR
2	41	+/+/+	doxorubicin/cyclophosphamide → paclitaxel/Herceptin	pCR
3	74	−/−/−	doxorubicin/cyclophosphamide → paclitaxel	Non-pCR
4	25	−/−/−	doxorubicin/cyclophosphamide → paclitaxel	Non-pCR
5	26	−/−/+	doxorubicin/cyclophosphamide → paclitaxel /Herceptin	pCR
6	41	−/−/−	Carboplatin/paclitaxel → doxorubicin/cyclophosphamide	pCR
7	37	−/−/−	Carboplatin/paclitaxel → doxorubicin/cyclophosphamide	Non-pCR
8	41	+/−/−	doxorubicin/cyclophosphamide → paclitaxel	pCR
9	47	+/+/−	doxorubicin/cyclophosphamide → paclitaxel	pCR
10	54	+/+/−	doxorubicin/cyclophosphamide → paclitaxel	Non-pCR
11	59	−/−/−	Pembrolizumab (or placebo)/Carboplatin/paclitaxel	Non-pCR
12	63	+/+/−	doxorubicin/cyclophosphamide → paclitaxel	Non-pCR
13	27	+/+/−	doxorubicin/cyclophosphamide → paclitaxel	Non-pCR
14	32	+/+/+	Taxotere/Carboplatin/Herceptin/Pertuzumab	Non-pCR
15	52	−/−/−	Carboplatin/paclitaxel → doxorubicin/cyclophosphamide	Non-pCR
16	38	−/−/−	Carboplatin/paclitaxel → doxorubicin/cyclophosphamide	pCR
17	38	−/−/−	Pembrolizumab (or placebo)/Carboplatin/paclitaxel	pCR
18	62	−/−/−	doxorubicin/cyclophosphamide → paclitaxel	Non-pCR
19	38	+/+/+	Taxotere/Carboplatin/Herceptin/Pertuzumab	Non-pCR
20	42	+/+/−	doxorubicin/cyclophosphamide → paclitaxel	Non-pCR
21	53	+/+/−	doxorubicin/cyclophosphamide → paclitaxel	Non-pCR
22	58	−/−/−	Pembrolizumab (or placebo)/Carboplatin/paclitaxel	Non-pCR
23	48	+/+/−	doxorubicin/cyclophosphamide → paclitaxel	Non-pCR
24	50	+/+/+	Taxotere/Carboplatin/Herceptin/Pertuzumab	Non-pCR
25	64	+/+/−	doxorubicin/cyclophosphamide → paclitaxel	Non-pCR
26	40	+/−/+	Taxotere/Carboplatin/Herceptin/Pertuzumab	pCR
27	31	−/−/−	doxorubicin/cyclophosphamide → paclitaxel	Non-pCR
28	54	−/−/−	Talazoparib	Non-pCR

### General study design

**Fig. 1:**
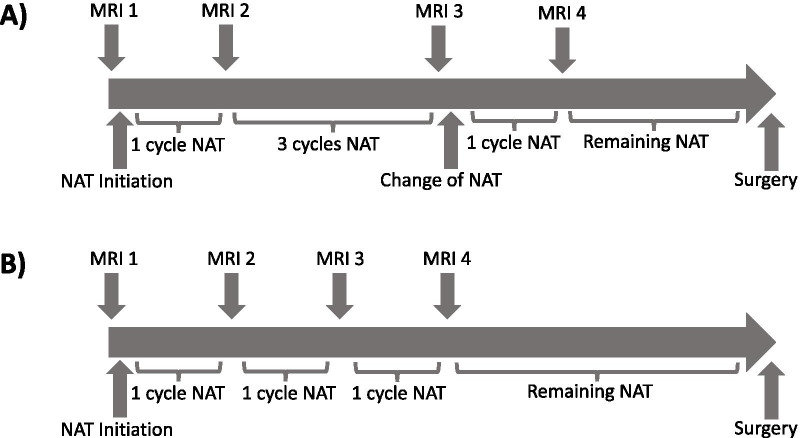
The diagram shows the timing of each MRI relative to the NAT regimen for patients who received two separate regimens (**A**) and patients who remained on a single regimen throughout the course of NAT (**B**)

Patients underwent quantitative MRI at one of two community imaging clinics (separate and in addition to standard-of-care imaging). In this study, a ‘community-care imaging center’ was defined as a non-academic, non-research setting (i.e., does not actively train any medical residents). Seven study participants received all imaging at a community hospital (not affiliated with an academic medical center) and the other 21 participants received all imaging at an outpatient imaging center. In the clinical workflow at these sites, the MRI technologists were directly involved and responsible for positioning the patients and deploying the research imaging protocols. Study personnel designed and installed the imaging protocol. Patients received a baseline session prior to beginning NAT (MRI 1) and following one round of NAT (MRI 2). For patients whose NAT included a second therapeutic regimen, MRIs 3 and 4 were performed prior to and after the first round of the second therapeutic regimen, respectively (Fig. 1A). For patients who maintained the same regimen throughout NAT, MRIs 3 and 4 were acquired after two and three rounds of NAT, respectively (Fig. 1B). Of the patients who enrolled in the study, three dropped out prior to MRI 2 and an additional two dropped out prior to MRI 3. None of these study dropouts achieved pCR. Additionally, no tumor was visible at MRI 2 for one patient who achieved pCR, no tumor was visible at MRI 3 for two patients who achieved pCR, and no tumor was visible at MRI 4 for three patients who achieved pCR. As there was no viable tumor on which to measure ADC or *K*^trans^ these measures were not performed at time points where tumor was no longer detectable.

### Image acquisition

MRI examinations were performed on a Siemens 3T Magnetom Skyra MR scanner (Siemens Medical Solutions USA, Malvern, PA) at both clinical community imaging facilities equipped with either an 8- or 16-channel receive double-breast coil (Sentinelle, Invivo, Gainesville, Florida). Image acquisition parameters are summarized in Table 2. DW-MRI was acquired using a monopolar single-shot spin-echo echo planar imaging (EPI) sequence with diagonal monopolar diffusion-encoding gradients. Six acquisitions were averaged for *b*-values of 0 and 200 s/mm^2^, while 18 acquisitions were averaged for the *b*-value of 800 s/mm^2^; this allowed for approximately equal signal-to-noise ratios [34] for all three *b*-values. DW-MRI was acquired over 10 sagittal slices centered on the tumor with 5 mm thickness and no slice gap. Spectrally selective adiabatic inversion recovery fat suppression was included for a total scan time of 1 min 39 s.

**Table 2:** MRI acquisition parameters (TR, repetition time; TE, echo time; FOV, field of view; GRAPPA, GeneRalized Autocalibrating Partial Parallel Acquisition)

MRI parameters	Anatomical scan	DW-MRI	*B*_1_ mapping	*T*_1_-mapping: variable flip angle	DCE-MRI
Scan sequence	*T*_1_-weighted 3D gradient-echo FLASH	Single-shot spin-echo (SE) echo planar (EPI)	3D spoiled gradient echo	*T*_1_-weighted 3D spoiled gradient echo	*T*_1_-weighted 3D spoiled gradient echo
*TR* (ms)	5.3	3000	8680	7.9	7.02
*TE* (ms)	2.3	52	2	2.4	4.6
Flip angle (°)	10	90	8	2, 4, 6, 8, 10, 12, 14, 16, 18, 20	6
Acquisition matrix	256 × 256	128 × 128	96 × 96	192 × 192	192 × 192
FOV (mm)	256 × 256	256 × 256	256 × 256	256 × 256	256 × 256
Slice thickness (mm)	1	5	5	5	5
GRAPPA acceleration factor	2	2	N/A	3	N/A
Fat suppression	SPAIR	SPAIR	N/A	N/A	N/A
Acquisition time (min:s)	3:11	1:39	0:34	0:50	8:00

The *T*_1_ map and DCE-MRI scans were acquired using a three-dimensional spoiled gradient-echo sequence over 10 sagittal slices with slice thickness of 5 mm. To construct a map of the longitudinal relaxation rate (*T*_1_), variable flip angle data with 10 flip angles (2, 4, …, 20) were acquired in a total scan time of 50 s. A Siemens TurboFLASH sequence was used to map the *B*_1_ field to correct for transmit inhomogeneity. Due to the inclusion of a slice gap in the *B*_1_ mapping protocol, two acquisitions were performed to cover the same field of view (FOV) as the *T*_1_ measurements for a total acquisition time of 34 s. DCE scans were acquired with a temporal resolution of 7.27 s for eight minutes. A catheter placed within an antecubital vein delivered a gadolinium-based contrast agent (0.1 mmol/kg of Multihance or 10 mL of Gadovist) at 2 mL/s followed by a 20 mL saline flush via a power injector after the acquisition of the first minute of dynamic scans which served as baseline. A separate high-resolution anatomical MRI was performed before and after contrast agent administration to aid manual tumor segmentation.

### Tumor segmentation

The tumor was first manually segmented on 2D slices with guidance by a certified fellowship-trained breast radiologist. This segmentation was used to calculate Response Evaluation Criteria in Solid Tumors (RECIST; [35]) measurements of longest tumor diameter. Following the manual segmentation, the tumor region of interest (ROI) was then automatically refined to detect enhancing tumor voxels using methods adapted from Giger et al*.* [36] and further described in our previous work [37] and Additional file 1: Methods.

### Image analysis

Prior to image analysis, all images for each patient from a single scan session were aligned to a common space via rigid registration (*imregtform*, MATLAB) to minimize patient motion.

The DW-MRI was used to extract ADC values (see Additional file 1: Methods for details) for every voxel within the tumor and the mean ADC within the tumor was calculated. Tumor cellularity at each voxel was calculated from ADC values as previously described [38] (see Additional file 1: Methods for details). To calculate cellularity at each voxel, we assume that a tumor voxel with the minimum ADC, ADC_min_, contains the maximum number of cells, while voxels with an ADC equivalent to free water, ADC_w_, are devoid of tumor cells. Then, by assuming an individual tumor cell has a volume of 4189 µm^2^ to derive the carrying capacity of each voxel, Θ, we approximate the total number of tumor cells within each voxel by computing Θ *[(ADC_w_ − ADC(*x*))/ADC_w_ − ADC_min_).

*T*_1_ maps were generated from the variable flip angle data along with a *B*_1_ map to correct for inaccuracies in the transmitted *B*_1_ field as is commonly seen in gradient-echo acquisitions at higher fields [39]. Details on this process are presented in Additional file 1: Methods.

The DCE-MRI data were analyzed using the Patlak model [40], which returns estimates of *K*^trans^ using the first 36.35 s (i.e., the first five post-contrast injection images) of the time course. Detailed description of this analysis is provided in Additional file 1: Methods. Due to evidence of non-normality in the voxel distribution of *K*^trans^, median tumor *K*^trans^ values were calculated. A ‘bulk tumor flow’ parameter (in units of [ml/min]) was also calculated as the product of the mean *K*^trans^ value of the tumor and the tumor volume.

### Pathological response

Histopathologic analysis was performed at study sites as standard of care and reported to the referring oncologist. pCR was the reference standard for determining response, defined as no residual invasive disease in either breast or axillary lymph nodes after NAT. Patients were categorized as having pCR or non-pCR on the basis of postsurgical histopathologic examination findings.

### Statistical analysis

Linear regression was performed to quantify changes in each MRI parameter over time across the entire cohort. Differences in baseline (MRI 1) parameters between the pCR and non-pCR cohorts were assessed by the Mann–Whitney test. The percent change in each imaging parameter relative to baseline was calculated by determining the relative change from MRI 1 to each subsequent MRI. Multiple Mann–Whitney tests were used to compare pCR versus non-pCR groups across the treatment time course. The Holm–Sidak method was used to adjust p values for multiple comparisons across time points. Differences between the pCR and non-pCR groups were considered significant when the adjusted p values were less than 0.05. Receiver operating characteristic (ROC) curves were generated for the relative change in each MRI parameter post baseline measurement. The ability of each parameter to discriminate between the pCR and non-pCR groups was estimated using the area under the ROC curve with corresponding 95% confidence interval. Statistical analysis was performed using Prism 8 software (Graphpad, San Diego, CA).

## Results

**Fig. 2:**
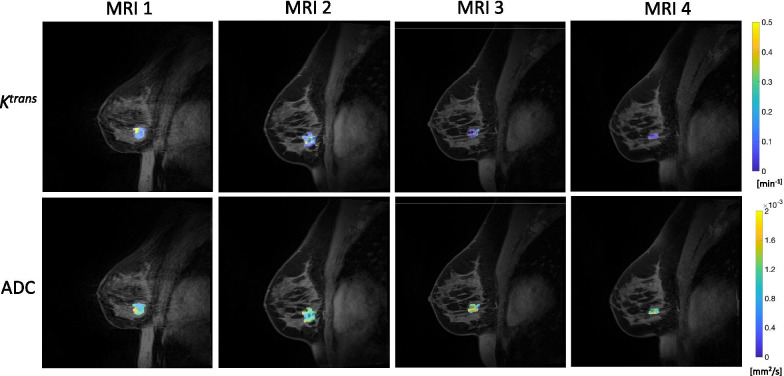
Representative *K*^trans^ (top) and ADC (bottom) maps over the course of therapy for a woman who achieved pCR (patient #9 in Table 1). Median tumor *K*^trans^ values decline over the course of therapy, from 0.12 min^−1^ at MRI 1 to 0.10 min^−1^ at MRI 2 to 0.02 min^−1^ at MRIs 3 and 4. Mean tumor ADC values increase over the course of therapy, from 0.0011 mm^2^/s at MRIs 1 and 2 to 0.0013 mm^2^/s at MRIs 3 and 4. For display purposes, *K*^trans^ and ADC parametric maps are interpolated to the resolution of anatomical images and overlaid on top of the anatomical images. (Note that all analysis was performed on the resolution at which the data were acquired.)

**Fig. 3:**
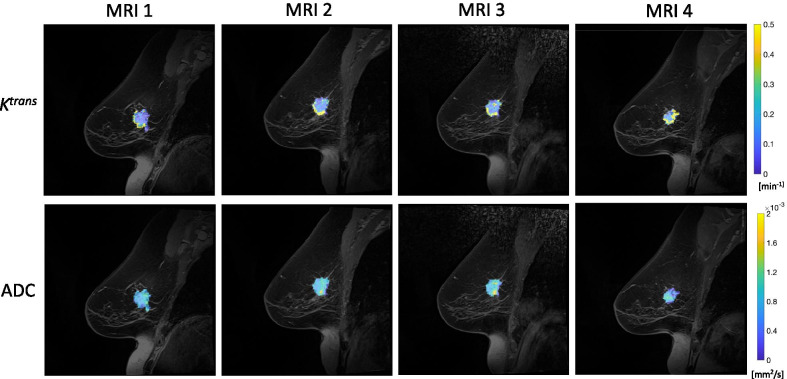
Representative *K*^trans^ (top) and ADC (bottom) maps over the course of therapy for a woman who had stable disease (patient #4 in Table 1). Median *K*^trans^ values increase over the course of therapy, from 0.10 min^−1^ at MRI 1 to 0.15 min^−1^ at MRI 2 and to 0.14 min^−1^ at MRIs 3 and 4. Mean tumor ADC values are largely unchanged over the course of therapy, from 0.0009 mm^2^/s at MRIs 1, 2, and 3 to 0.0008 mm^2^/s at MRI 4. For display purposes, *K*^trans^ and ADC parametric maps are interpolated to the resolution of anatomical images and overlaid on top of the anatomical images. (Note that all analysis was performed on the resolution at which the data were acquired.)

Prior to the start of NAT, there were no significant differences in any quantitative MRI parameters calculated at baseline (MRI 1) between patients who achieved pCR and those who did not. Additionally, there were no significant differences in any quantitative MRI parameters across the two study sites. After commencing NAT, we observed declines in tumor volume and median *K*^trans^ and increases in mean tumor ADC in women who ultimately achieved pCR, as shown in Fig. 2 for a representative study participant who achieved pCR. In contrast, women who did not achieve pCR did not consistently demonstrate declines in either tumor size or *K*^trans^ nor increases in ADC, as shown for a representative study participant who had stable disease (Fig. 3).

**Fig. 4:**
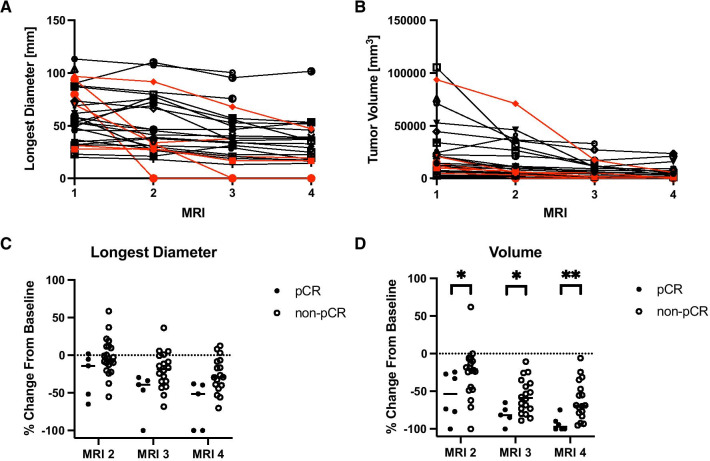
Tumor longest diameter (**A**) and tumor volume (**B**) tend to decline over the course of NAT. Patients who achieved a pCR are shown in red in Panels A and B. No significant differences in the relative change in tumor longest diameter from baseline measurement at MRI 1 (**C**) were observed between patients who achieved pCR and those who did not achieve pCR. Significant differences in tumor volume changes from baseline (**D**) were observed between patients who achieved pCR and those who did not achieve pCR at all three MRIs performed during the course of therapy (*p < 0.05; **p < 0.01; ***p < 0.001)

Tumor longest diameter (Fig. 4A) and volume (Fig. 4B) tended to decline over the course of NAT across the cohort of 28 study participants (*p* < 0.005). However, women who achieved pCR had greater (*p* < 0.05) relative declines in tumor volume at MRI 2 relative to pre-treatment values than those who did not ultimately achieve pCR (31 ± 15% greater decline in the pCR group). Additionally, the relative decline in tumor volume was significantly greater in the pCR group at each MRI performed during NAT (*p* < 0.05, Fig. 4D). In contrast, the relative decline in tumor longest diameter was not statistically different between the pCR and non-pCR cohorts at any MRI performed during NAT (Fig. 4C).

**Fig. 5:**
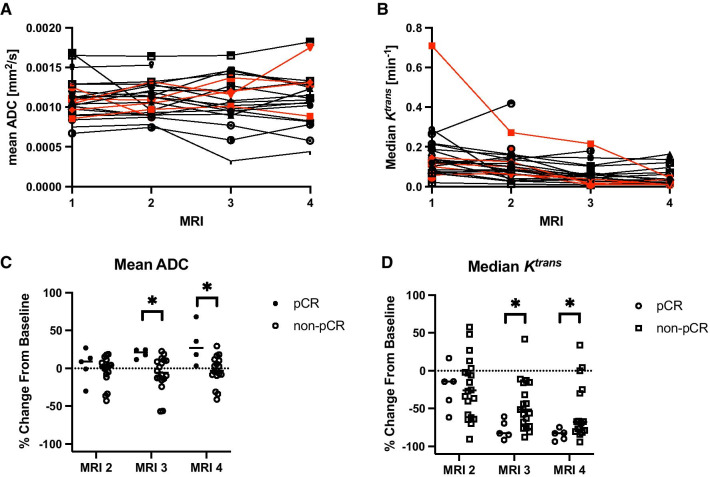
Mean tumor ADC (**A**) is relatively stable over the course of NAT while the median tumor *K*^trans^ (**B**) tends to decline over the course of NAT. Patients who achieved a pCR are shown in red in Panels A and B. Significant differences in the relative change in ADC from baseline (**C**) were observed between patients who achieved pCR and those who did not achieve pCR at the third and fourth MRI. Significant differences in the relative change in *K*^trans^ from baseline (**D**) were also observed between patients who achieved pCR and those who did not achieve pCR at the third and fourth MRI (*p < 0.05; **p < 0.01; ***p < 0.001)

Across the entire patient cohort, the mean tumor ADC displayed no significant change over the course of NAT (Fig. 5A). In contrast, the median *K*^trans^ significantly declined over the course of NAT (*p* < 0.005, Fig. 5B) across all patients. Changes in mean tumor ADC from baseline revealed significant differences between the pCR and non-pCR groups, with increases in the ADC in the pCR group of 21 ± 12% and 32 ± 14% at MRIs 3 and 4, respectively (*p* < 0.05, Fig. 5C). Similarly, the relative decline in median tumor *K*^trans^ was 32 ± 15% greater in the pCR group than the non-pCR group at MRI 3 and 31 ± 17% greater at MRI 4 (*p* < 0.05, Fig. 5D).

**Fig. 6:**
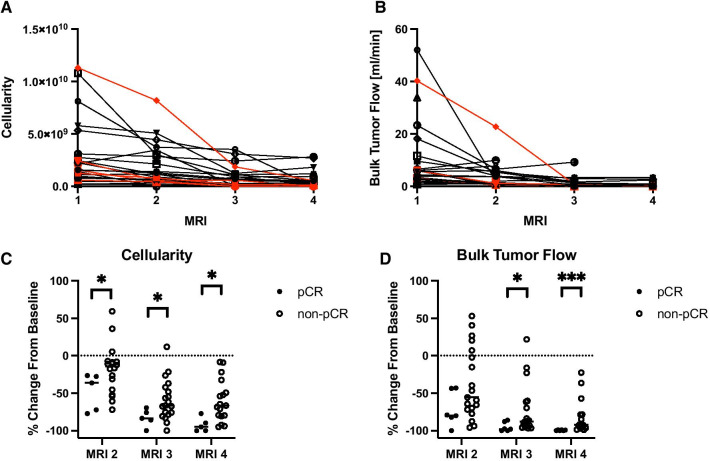
Total tumor cellularity (**A**) and bulk tumor flow (**B**) tend to decline over the course of NAT. Patients who achieved a pCR are shown in red in **A** and **B**. Significant differences in the relative change in cellularity from baseline **C** were observed between patients who achieved pCR and those who did not achieve pCR at all three MRIs following baseline. Significant differences in the relative change in bulk tumor flow from baseline **D** were observed between patients who achieved pCR and those who did not achieve pCR at the third and fourth MRI (*p < 0.05; **p < 0.01; ***p < 0.001)

Tumor cellularity (Fig. 6A) and bulk tumor flow (Fig. 6B) tended to decline over the course of NAT across all study participants (*p* < 0.005). The decline in tumor cellularity from baseline was 30 ± 16% greater in the pCR group at MRI 2, 24 ± 13% greater at MRI 3, and 33 ± 13% greater at MRI 4. This decline was significantly greater in the cohort who achieved pCR than the non-pCR cohort at all MRIs performed during NAT (*p* < 0.05, Fig. 6C). Likewise, the relative decline in bulk tumor flow from baseline was significantly greater in the pCR group at MRIs 3 and 4 (22 ± 14% greater at MRI 3 and 19 ± 10% greater at MRI 4, *p* < 0.05, Fig. 6D).

The diagnostic accuracy of each MRI parameter for predicting pCR was quantified using ROC analysis. The area under each ROC curve (AUC-ROC) is shown in Table 3 with ROC curves shown in Additional file 2: Fig. S1. Change in tumor volume from baseline was the best early predictor of pCR in breast tumors at MRI 2 (AUC-ROC = 0.82), change in mean ADC was the best predictor at MRI 3 (AUC-ROC = 0.93), and bulk tumor flow was the best predictor at MRI 4 (AUC-ROC = 1.00). In general, the predictive accuracy of each MRI parameter increased at later time points.

**Table 3:** Area under the receiver operating characteristic curve (AUC-ROC) for predicting pCR from different MRI measurements

	MRI 2	MRI 3	MRI 4
Longest diameter	0.71 [95% CI 0.46–0.97]	0.80 [95% CI 0.61–0.98]	0.84 [95% CI 0.66–1.00]
Tumor volume	0.82 [95% CI 0.65–0.99]	0.82 [95% CI 0.64–1.00]	0.88 [95% CI 0.73–1.00]
Mean ADC	0.58 [95% CI 0.24–0.91]	0.93 [95% CI 0.81–1.00]	0.88 [95% CI 0.71–1.00]
Median *K*^trans^	0.53 [95% CI 0.28–0.78]	0.85 [95% CI 0.69–1.00]	0.84 [95% CI 0.65–1.00]
Cellularity	0.81 [95% CI 0.62–1.00]	0.83 [95% CI 0.65–1.00]	0.88 [95% CI 0.73–1.00]
Bulk tumor flow	0.73 [95% CI 0.50–0.95]	0.81 [95% CI 0.59–1.00]	1.00 [95% CI 1.00–1.00]

Each column indicates the relative change in each parameter from the baseline MRI (i.e., MRI 1) at the specified MRI scan

## Discussion

Moving advanced technology from the research-oriented, academic medical setting into the community-based, standard-of-care setting has the potential to increase the level of care for the overwhelming majority of cancer patients. In particular, quantitative DCE- and DW-MRI have emerged as two MRI techniques that have matured to the point where they can provide accurate predictions of the response of breast tumors to NAT. However, there has been limited progress implementing these quantitative MRI methods for predicting response to NAT in the community care setting. Hurdles to community implementation include technical limitations porting quantitative protocols onto MRI scanners not governed by research agreements, differences in clinical and research workflows, exam scheduling, and data transfer. We demonstrate that these challenges can be overcome. We believe this represents an important first step to disseminating quantitative imaging beyond academic and research hospitals to local and regional imaging facilities. This has the potential to greatly expand the populations of breast cancer patients that have access to (1) advanced imaging and (2) participation in clinical trials that require advanced imaging.

Previous studies have shown that DCE- and DW-MRI can predict pathological response to NAT in locally advanced breast cancer. For example, quantitative pharmacokinetic DCE-MRI performed following one cycle of NAT showed that the parameters *K*^trans^ and *k*_*ep*_ (i.e., the intravasation rate constant) were excellent predictors of pCR prior to any significant changes in RECIST [23, 29, 41]. Likewise, measurements of ADC have shown that both baseline and changes in ADC can be better predictors of tumor response than measurements of tumor size [24, 28, 42–44] and can be further strengthened by breast cancer subtype stratification [45, 46]. Combining biological information from DCE- and DW-MRI further increases the predictive ability of quantitative MRI in the neoadjuvant setting, achieving AUC-ROCs between 0.88 and 0.92 [23, 47]. While these studies show positive advancements, they have yet to extend beyond the academic setting, thereby leaving an important gap in distributing this emerging technology to all patients.

Our study demonstrates that the predictive ability of MRI metrics acquired in the community setting is similar to those in an academic center. In particular, we found an AUC-ROC of 0.82 after only 1 cycle of NAT using semi-automatic measurements of tumor volume. This study further introduces a novel metric we term ‘bulk tumor flow’ that quantitatively describes the total blood flow to the tumor. Bulk tumor flow, which combines the automated method for extracting vascularized tumor volume and the mean tumor *K*^trans^, provided the highest overall AUC-ROC in this study, namely a perfect AUC of 1.0 at MRI 4, which was acquired at an average of 12 weeks into NAT across the cohort. Of note, the longest tumor diameter (which is used in RECIST for evaluating tumor response to therapy) was not able to predict pCR at any time point examined in this study. Typically, RECIST is implemented during clinical trials of highly selected patient populations receiving limited therapeutic regimens. Differences between clinical trial and ‘real world’ effectiveness have been well established [48]. This ‘real world’ effectiveness study of community-implemented quantitative MRI demonstrates that the MRI metrics investigated in this study may be more accurate than RECIST at predicting pCR across a wide range of treatments and subtypes of locally advanced breast cancer.

There are a number of limitations to this study. The same contrast agent was not used for all patients; though, each individual patient did receive the same contrast agent at all of their MRI exams. We note that this was necessary to work within multiple community imaging centers as the contrast agent and dose administered was based on site-specific protocols, and that image processing accounted for differences in contrast agent. Additionally, the treatment regimens differed between patients, as guided by the treating oncologists. While this makes the study more challenging to control for variation, it is exactly the type of patient population that community-based oncologists encounter on a daily basis and, therefore, has great practical relevance. Another area that requires further exploration for true widespread adoption is in the area of automated data analysis. We believe that the automated segmentation implemented to assess these predictions can be incorporated into routine clinical workflow; however, that has not been evaluated at this point. In this study, clinical radiologists were involved in the segmentation process, while imaging scientists conducted all quantitative analyses. An additional challenge associated with this study is the ability to conduct a protocol that works within the confines of non-research clinical scanners and within the time frame required by radiology centers performing breast MRIs. Finally, patient recruitment can be especially challenging in a non-academic environment where the oncologists, radiologists, and surgeons are not housed in one location; thus, the present study is limited to 28 patients.

## Conclusion

To the best of our knowledge, this represents the first study to show that quantitative DCE- and DW-MRI can be successfully implemented in community care facilities to accurately predict the response of locally advanced breast cancer to NAT. We showed that parameters characterizing the change in tumor volume, cellularity (ADC), and vascular characteristics (*K*^trans^) can predict pathological complete response to NAT significantly better than the RECIST criteria. Furthermore, integrating DW-MRI and DCE-MRI with semi-automated measurements of tumor volume further increases the ability to accurately predict response early in the course of therapy. By moving these emerging measures to community-based medical centers, where the majority of patients receive their care, we are dramatically increasing the patient population that can be served by advanced imaging.

## Supplementary Information

**Additional file 1**. Additional details describing image analysis methods.

**Additional file 2: Figure S1**. Receiver operating characteristic curves for each MRI parameter for predicting pCR from the relative change from baseline at each serial MRI.

## Data Availability

The imaging data used and/or analyzed in this study are available from the corresponding author upon reasonable request. All authors read and approved the final manuscript.
