# Supplementary material for: Quantitative multiparametric MRI predicts response to neoadjuvant therapy in the community setting
Source: Breast Cancer Res. 2021 Nov 27;23:110. doi: 10.1186/s13058-021-01489-6 (PMC8627106; doi:10.1186/s13058-021-01489-6)
Supplement: Supplementary file 1 — Additional file 1. Additional details describing image analysis methods. [file 13058_2021_1489_MOESM1_ESM.docx]

**ADDITIONAL FILE 1: METHODS**

**Tumor Segmentation**

Using the signal intensity time course from the DCE-MRI data as features of each voxel, fuzzy c-means clustering (*fcm*, MATLAB, The Mathworks, Natick, MA) was applied to the ROI to assign each voxel as either tumor or non-tumor. After this binary mask was determined, a region filling algorithm (*imfill*, MATLAB) was employed to generate a contiguous volume within the tumor mask. The tumor volume was then calculated by multiplying the number of voxels segmented into the lesion class by the voxel volume.

**Image Analysis**

ADC maps were calculated from the DW-MRI data by fitting the data to Eq. [1]:

$S(b)=S_{0}\bullet\exp(-ADC\bullet b)$, [1]

where *S*(*b*) is the signal intensity in the presence of diffusion gradients at strength *b*, *S_0_* is the signal intensity in the absence of diffusion gradients, and *b-*value is the strength of the diffusion gradients. Eq. [1] was fit to the signal intensities from the 200 and 800 s/mm^2^ *b*-values on a voxel-by-voxel basis using a non-linear least squares approach (*lsqnonlin*, MATLAB, The MathWorks, Natick, Massachusetts). Mean ADC of the tumor was calculated by summing the ADC value at each voxel and dividing by the number of tumor voxels.

Cellularity was calculated each voxel according to Eq. [2]:

$N\left( t \right)=\cdot\left\lfloor\frac{{ADC}_{W}-{ADC}_{t}}{{ADC}_{W}-{ADC}_{min}} \right\rfloor,$[2]

where N(t) is the number of tumor cells. We assume that a tumor voxel with the minimum ADC, ADC_min_, contains the maximum number of cells, while voxels with an ADC equivalent to free water, ADC_w_, are devoid of tumor cells. We assume an individual tumor cell has a volume of 4189 µm^2^ to derive the carrying capacity of each voxel, Θ.

Quantitative *B_1_*-corrected, variable flip angle, *T_1_* values were calculated for each voxel by fitting the signal intensity, *S*, data to Eq. [2]:

$S=S_{0}\bullet\left[\frac{\sin(f\bullet\alpha)\bullet(1-\exp\left(-\frac{TR}{T_{1}}\right))}{1-(\exp\left(-\frac{TR}{T_{1}}\right)\bullet\cos(f\bullet\alpha))}\right]$ , [3]

where *S_0_* is a constant related to scanner gain and proton density, *α* is the prescribed flip angle, and *f* is the flip angle correction factor that accounts for inhomogeneity in *B_1_*. We have also assumed *TE* << *T_2_^*^*.

For DCE-MRI analysis, the Patlak model was defined as:

$C_{t}\left( t \right)= K^{trans}\int_{0}^{t} C_{p}(t')dt'$ [4]

where *C_t_*(*t*) is the concentration of contrast agent in the tissue as a function of time in units of mM, *C_p_*(*t*) is the concentration of contrast agent in the blood plasma (i.e., the arterial input function as estimated from the axillary artery) in units of mM, and *K^trans^* is the volume transfer constant in units of mL (blood)/ mL (tissue) /min. As the Patlak model assumes no intravasation from the tissue to the vessel occurs in the early moments after the injection of the contrast agent, we excluded the washout phase and fit the Patlak model to only the enhancement portion of the signal intensity curves, namely the first five post-injection acquisitions (i.e., 36.35 seconds). The Patlak model was fit to the data using the *lsqnonlin* function in MATLAB to estimate *K^trans^* at each voxel within the tumor ROI. Voxels for which the estimated parameters fell outside of the physiological range (i.e., 0.001 < *K^trans^* < 5.0 min^-1^) were eliminated from further analysis.

**ADDITIONAL FILE FIGURE LEGND**

**Additional file 1: Figure S1.** Receiver operating characteristic curves for each MRI parameter for predicting pCR from the relative change from baseline at each serial MRI.
