# Supplementary figures and images for: Quantitative multiparametric MRI predicts response to neoadjuvant therapy in the community setting
Source: Breast Cancer Res. 2021 Nov 27;23:110. doi: 10.1186/s13058-021-01489-6 (PMC8627106; doi:10.1186/s13058-021-01489-6)

MRI 4

## Bulk Tumor Flow

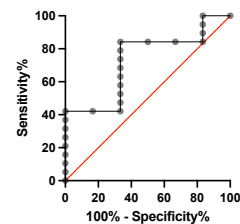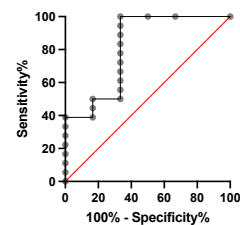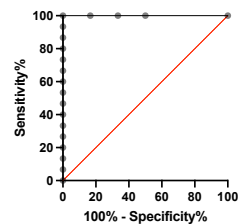

Supplement: Supplementary file 2 — Additional file 2: Figure S1. Receiver operating characteristic curves for each MRI parameter for predicting pCR from the relative change from baseline at each serial MRI. [file 13058_2021_1489_MOESM2_ESM.pdf]
